# Curriculum Vitae Workshops: Promoting Transparency in Alpha Omega Alpha While Serving Student Needs

**DOI:** 10.1177/23821205211044604

**Published:** 2021-12-23

**Authors:** Christina Dorismond, Joshua S. Ellis, Maureen D. Rosen, Shweta Bhatia, Ryan Searcy, Lauren K. Leeper, Sheryl G. Jordan

**Affiliations:** 1 Vanderbilt University Medical Center, South Nashville, TN, USA; 2 Massachusetts General Hospital, Boston, MA, USA; 3 University of North Carolina School of Medicine, Chapel Hill, NC, USA

**Keywords:** Alpha Omega Alpha, professional development, curriculum vitae, medical students

## Abstract

**OBJECTIVE:**

To promote equity in the Alpha Omega Alpha Honor Medical Society (AOA) selection process, clear and timely communication of eligibility criteria is needed. Herein, the authors describe and assess the effectiveness of a novel method for improving transparency in the AOA selection process while also teaching students key professional development skills.

**METHODS:**

The authors hosted curriculum vitae (CV) workshops for interested medical students. One part of each session was dedicated to sharing information about AOA and its selection process, while the rest focused on teaching students how to build effective CVs. After the most recent session, students were asked to complete a survey about the effectiveness of the workshop.

**RESULTS:**

Between 2019 and 2020, three CV workshops were hosted. Interest in the events was high, with approximately 15 to 30 first- and second-year medical students participating in each. Based on survey results, participants found the workshop helped them gain a better understanding of AOA eligibility and selection (100%, *n* = 10) and taught them key CV development skills (100%, *n* = 10).

**CONCLUSION:**

These workshops are a novel approach to disseminating AOA eligibility criteria and can be employed by medical schools to promote transparency in the AOA selection process. They also give students the skills to craft CVs that will better prepare them for applying to residency and other academic opportunities. As such medical schools and AOA chapters should consider implementing a similar model at their institutions.

## Background

Founded in 1902, Alpha Omega Alpha Honor Medical Society (AOA) has sought to honor individuals who have demonstrated “a lasting commitment to professionalism, leadership, scholarship, research, and community service.”^1^ Since its inception, more than 200 000 medical students, residents, fellows, faculty, and alumni have been inducted into the organization. Membership in AOA has been shown to be a noteworthy and valuable achievement for many medical students, with several studies showing that election to the honor society is associated with an increased likelihood of receiving a residency interview offer, matching into the student's preferred residency program, and pursuing a career in academic medicine.^2–4^

In recent years, however, there have been growing concerns regarding equity in AOA selection, particularly for those underrepresented in medicine (URM), which is defined by the Association of American Medical Colleges as “those racial and ethnic populations that are underrepresented in the medical profession relative to their numbers in the general population.”^5^ Studies have shown that AOA membership is 6 times greater for White students than for Black students and 2 times greater for White students than for Asian students, despite controlling for relevant academic factors.^6,7^

Medical schools across the nation have sought to address these disparities in AOA membership in a number of ways. Some medical schools have chosen to suspend AOA selection entirely, while others have opted to modify the selection criteria for their cohort of eligible or nominated AOA students to create a more equitable process.^8,9^ At our institution, we implemented a holistic evaluation of students nominated for AOA membership. We decreased our emphasis on academic class rankings and numerical grades, and instead began to emphasize students’ research, leadership, and service based on the information submitted by students in their curriculum vitae (CV). We also began publishing nomination and selection criteria on our website and disseminated clear timelines regarding the nomination and election process prior to each election. Moreover, to give students the skills needed to build their CVs and address any lack of knowledge about AOA that may disadvantage certain students, we began hosting information sessions about the honor medical society in combination with CV-building workshops.

Recently, the Board of Directors of AOA removed the requirement that students be in the top 25% of their class to be nominated for AOA and also increased the number of graduating students who could be elected to the society.^10^ This new policy gives medical schools and their AOA chapters the opportunity to develop new methods for determining eligibility and to move away from a reliance on clinical clerkship grades which tend to disadvantage URM students.^11,12^

As medical schools begin to develop new criteria for eligibility, it is imperative that information regarding the new criteria is shared clearly with students at all levels of medical school. A lack of understanding of how students become eligible and are elected to AOA may be disadvantageous to some students, preventing them from putting their best foot forward for AOA selection and unfairly benefiting those who have a previous understanding of AOA and what is needed for membership. Thus, there is a strong need for early and transparent information-sharing regarding AOA. Herein, we describe how our institution utilized CV workshops to improve transparency in our AOA selection process and provided students with key professional development skills. This can serve as a model for other medical schools as they develop new criteria for selection.

## Methods

Between 2019 and 2020, we hosted 3 CV workshops for interested medical students. Each session was advertised to all 180 first-year medical students and all 180 second-year medical students via email to each class's listserv. Prior to each event in 2019, students were asked to sign-up for the sessions due to space limitations. In 2020, the sessions were held virtually due to COVID-19. The workshops were led by the AOA chapter presidents and vice presidents and several faculty members. Each hour-long session began with a short presentation about AOA and its selection process. We then transitioned to CV development training during which we modeled ways in which students could highlight their scholarship, leadership, service, extracurricular activities, and research in a professional standardized CV format. We also shared information about the residency application process and tips on how to build a CV in a way that could be easily translated to residency applications during the appropriate application cycle. After the event in the Fall of 2020, attendants of that session were asked to complete an Institutional Review Board-approved survey to evaluate the effectiveness of the CV workshop in improving their CV-building skills and in increasing their knowledge about AOA. Those who completed the survey provided written informed consent prior to participation. Survey results were analyzed with descriptive statistics via Qualtrics (Provo, UT).

## Results

A total of ∼75 students, ~30 for each session in 2019 and ~15 for the session in 2020, participated in the workshops, with most attendees being first- and second-year medical students. During each workshop, we directly addressed student questions and misconceptions regarding AOA. We then led them through how to build effective CVs and answered their questions. Participant verbal feedback following each session was uniformly positive.

Of the 15 participants from the session in 2020, 10 completed the post-workshop survey, including 5 first-year students and 5 second-year students. Following the session, all survey participants (*n* = 10, 100%) agreed or strongly agreed that they had a better understanding of how students become eligible for AOA and how students are ultimately elected to AOA (Table 1). All 10 also agreed that they had a better understanding of the activities to include in their CV (100%) and how to format their CVs (100%). The majority also felt better prepared to apply to leadership, research, scholarship, or other academic opportunities (*n* = 9, 90%) and to apply to residency (*n* = 8, 80%) after attending the workshop.

**Table 1.** CV workshop survey responses.

STATEMENT	STRONGLY AGREE	AGREE	NEITHER AGREE NOR DISAGREE	DISAGREE	STRONGLY DISAGREE
I have a better understanding of how students become eligible for AOA.	5 (50%)	5 (50%)	0 (0%)	0 (0%)	0 (0%)
I have a better understanding of how students are ultimately elected to AOA.	5 (50%)	5 (50%)	0 (0%)	0 (0%)	0 (0%)
I have a better understanding of the activities to include in my CV.	8 (80%)	2 (20%)	0 (0%)	0 (0%)	0 (0%)
I have a better understanding of how to format my CV.	6 (60%)	4 (40%)	0 (0%)	0 (0%)	0 (0%)
I feel better prepared to apply to leadership, research, scholarship, or other academic opportunities.	4 (40%)	5 (50%)	1 (10%)	0 (0%)	0 (0%)
I feel better prepared to apply to residency	3 (30%)	5 (50%)	2 (20%)	0 (0%)	0 (0%)
I would recommend this workshop to a friend.	6 (60%)	4 (40%)	0 (0%)	0 (0%)	0 (0%)

Abbreviations: AOA: Alpha Omega Alpha Honor Medical Society; CV: curriculum vitae.

## Discussion

Given recent changes to the AOA selection process, it is likely that many medical schools will need to develop new methods for student nomination to AOA. In so doing, it is critical that changes are relayed clearly and in a timely manner to students. Workshops such as the ones presented here give AOA chapters an opportunity to share information regarding their organization and their eligibility or election process. This program is also a key step toward reducing the advantage that some students may have over others during the AOA selection, namely knowing how to build and deliver an effective CV. Additionally, during the workshops, students learn ways to highlight their accomplishments outside of academics which can facilitate a more holistic review of students during the AOA elections and lead to a more equitable process.

While only a select group of medical students will ultimately be nominated for AOA, nearly all will apply to residency programs and many will also apply for other academic opportunities. A significant portion of the application to residency relies on the Electronic Residency Application Service reporting of students’ awards, research, leadership, volunteering, and work experiences in much the same way they are presented on a CV.^13^ As such, better preparation of CVs early in medical school can improve and streamline the residency application process for all students, including those who are not selected for AOA membership. Furthermore, as many scholarship, leadership, and academic opportunities rely on CVs as part of their application process, improving a student's CV can improve their chances of selection for these opportunities. Our study suggests that these workshops maybe effective in accomplishing these goals. Thus, CV workshops have the added advantage of better preparing all students, including those not nominated for AOA, to apply for residency and other opportunities and should be considered at all medical schools.

As an AOA chapter, we plan to continue to host CV workshops to reduce misconceptions about AOA, improve knowledge about the honor medical society's selection process, and help students build more effective CVs. As the students who participate in our workshops become eligible for AOA, we will complete an analysis of their effectiveness long-term. In the meantime, we share this innovative idea as a model for other institutions on how to improve knowledge about AOA and its selection process in light of the recent changes to the AOA nomination procedure.
